# Anti-Atopic Dermatitis Effects of Abietic Acid Isolated from Rosin under Condition Optimized by Response Surface Methodology in DNCB-Spread BALB/c Mice

**DOI:** 10.3390/ph16030407

**Published:** 2023-03-07

**Authors:** Jumin Park, Ji Eun Kim, You Jeong Jin, Yu Jeong Roh, Hee Jin Song, Ayun Seol, So Hae Park, Sungbaek Seo, Heeseob Lee, Dae Youn Hwang

**Affiliations:** 1 Department of Food Science and Nutrition, College of Human Ecology, Pusan National University, Busan 46241, Republic of Korea; 2 Department of Biomaterials Science (BK21 FOUR Program), Life and Industry Convergence Research Institute, College of Natural Resources and Life Science, Pusan National University, Miryang 50463, Republic of Korea; 3 Longevity & Wellbeing Research Center, Laboratory Animals Resources Center, College of Natural Resources and Life Science, Pusan National University, Miryang 50463, Republic of Korea

**Keywords:** abietic acid, atopic dermatitis, RSM, LPS, RAW264.7 macrophages, DNCB

## Abstract

Abietic acid (AA) is known to have beneficial effects on inflammation, photoaging, osteoporosis, cancer, and obesity; however, its efficacy on atopic dermatitis (AD) has not been reported. We investigated the anti-AD effects of AA, which was newly isolated from rosin, in an AD model. To achieve this, AA was isolated from rosin under conditions optimized by response surface methodology (RSM), and its effects on cell death, iNOS-induced COX-2 mediated pathway, inflammatory cytokine transcription, and the histopathological skin structure were analyzed in 2,4-dinitrochlorobenzene (DNCB)-treated BALB/c mice after treatment with AA for 4 weeks. AA was isolated and purified through isomerization and reaction-crystallization under the condition (HCl, 2.49 mL; reflux extraction time, 61.7 min; ethanolamine, 7.35 mL) established by RSM, resulting in AA with a purity and extraction yield of 99.33% and 58.61%, respectively. AA exhibited high scavenging activity against DPPH, ABTS, and NO radicals as well as hyaluronidase activity in a dose-dependent manner. The anti-inflammatory effects of AA were verified in lipopolysaccharide (LPS)-stimulated RAW264.7 macrophages through amelioration of the inflammatory response, including NO production, iNOS-induced COX-2 mediated pathway activation, and cytokine transcription. In the DNCB-treated AD model, the skin phenotypes, dermatitis score, immune organ weight, and IgE concentration were significantly ameliorated in the AA cream (AAC)-spread groups compared to the vehicle-spread group. In addition, AAC spread ameliorated DNCB-induced deterioration of skin histopathological structure through the recovery of the thickness of the dermis and epidermis and the number of mast cells. Furthermore, activation of the iNOS-induced COX-2 mediated pathway and increased inflammatory cytokine transcription were ameliorated in the skin of the DNCB+AAC-treated group. Taken together, these results indicate that AA, newly isolated from rosin, exhibits anti-AD effects in DNCB-treated AD models, and has the potential to be developed as a treatment option for AD-related diseases.

## 1. Introduction

Abietic acid (AA, C_20_H_30_O_2_, MW 302.458 g/mol) is an organic acid naturally found in pine obtained from *Pinus* sp. (Pinaceae). It belongs to the abietane diterpene group of organic compounds derived from four isoprene units (20 carbon atoms) [1,2]. Natural AA and its derivatives with high anti-oxidant activity have received great attention due to its various therapeutic effects, including anti-microbial, anti-ulcer, anti-cardiovascular, and anti-allergy activities [3,4,5,6,7,8]. However, the isolation of natural AA from rosin, which a solid form of resin collected from pines and other plants, has only been attempted in several studies through isomerization, amination, and recrystallization processes [9,10,11]. Among these, an early study isolated this compound without reporting accurate yield or purity and only used it for efficacy evaluation [9]. Only recent studies have reported actual values for the purity and yield of AA. Approximately 98 g of AA was first isolated from 250 g of wood rosin at a yield of 39.2% [10]. In addition, AA with high purity (98.52%) was obtained from gum rosin with a yield of 54.93% [11]. To date, the purification stage of AA involves a complex process and has low yields, although the isolation stage consists of a similar process. Additionally, the optimization of AA isolation conditions using response surface methodology (RSM) has never been attempted. Therefore, it is necessary to optimize and simplify the isolation and purification stages to improve the yield and purity of natural AA.

The beneficial effects of AA have been actively studied in various biological fields, including angiogenesis, cancer, nephropathy, lung injury, osteolysis, endoplasmic reticulum stress (ER stress), and inflammation. Among them, notable suppression effects were detected in the growth, metastasis, and invasiveness of several types of cancer cells, such as melanoma, non-small-cell lung and breast cancer cells, and breast cancer cells [12,13,14]. In addition, AA accelerates cutaneous wound healing by enhancing angiogenesis [15], ameliorating sepsis-induced lung injury by inhibiting the nuclear factor-kappa B (NF-κB) signaling pathway [16], and mitigating obesity in models related to suppressing steatosis and adipogenesis [17,18]. The osteoprotective effect of AA was detected in receptor activator of nuclear factors κB ligand (RANKL)-induced osteoclastogenesis and in lipopolysaccharide (LPS)-induced osteolysis models [19], while its mitigation effects on nephropathy progression were detected in the kidney in type-2 diabetes mellitus (DM) [1]. Furthermore, AA significantly ameliorated psoriasis-like skin inflammation in imiquimod (IMQ)-treated BALB/c mice, interleukin (IL)-1β-induced inflammation in human osteoarthritic chondrocytes, airway inflammation in an allergic asthma model, and kidney inflammation in a type-2 DM model [1,20,21,22]. However, whether AA has anti-atopic dermatitis (AD) effects in the DNCB-induced AD model remains unclear.

In this study, we investigated the anti-AD activity of AA newly isolated from rosin and its molecular mechanism of action in DNCB-treated BALB/c mice with AD phenotypes based on the results of LPS-stimulated RAW264.7 macrophages.

## 2. Results

### 2.1. Isolation and Purification of AA under Optimal Condition Established by RSM, and Its Characterization

Firstly, an optimal condition for AA isolation was established by RSM to improve the purity and yield of AA. According to the RSM design, 17 combinations were performed in triplicate and the obtained results are showed in Supplementary Table S1. During the optimization, the response results in the following regression equations:

Y_1_ = 60.00 + 1.91X_1_ + 1.19X_2_ − 1.40X_3_ − 0.90X_1_X_2_ + 0.47X_1_X_3_ − 1.77X_2_X_3_ + 2.14X_1_^2^ − 1.37X_2_^2^ − 1.50X_3_^2^

Y_2_ = 90.27 + 1.26X_1_ + 1.17X_2_ − 1.31X_3_ − 1.11X_1_X_2_ + 0.67X_1_X_3_ − 1.56X_2_X_3_ + 1.60X_1_^2^ − 1.43X_2_^2^ − 1.45X_3_^2^

where Y_1_ and Y_2_ are the maximal extraction yield and purity of AA as a function of the amount of HCl (X_1_), reflux extraction time (X_2_), and amount of ethanolamine (X_3_). Based on the experimental response, the optimized conditions based on both extraction yield and purity were determined as the amount of HCl added (2.49 mL), reflux extraction time (61.7 min), and the amount of ethanolamine added (7.35 mL).

**Figure 1:**
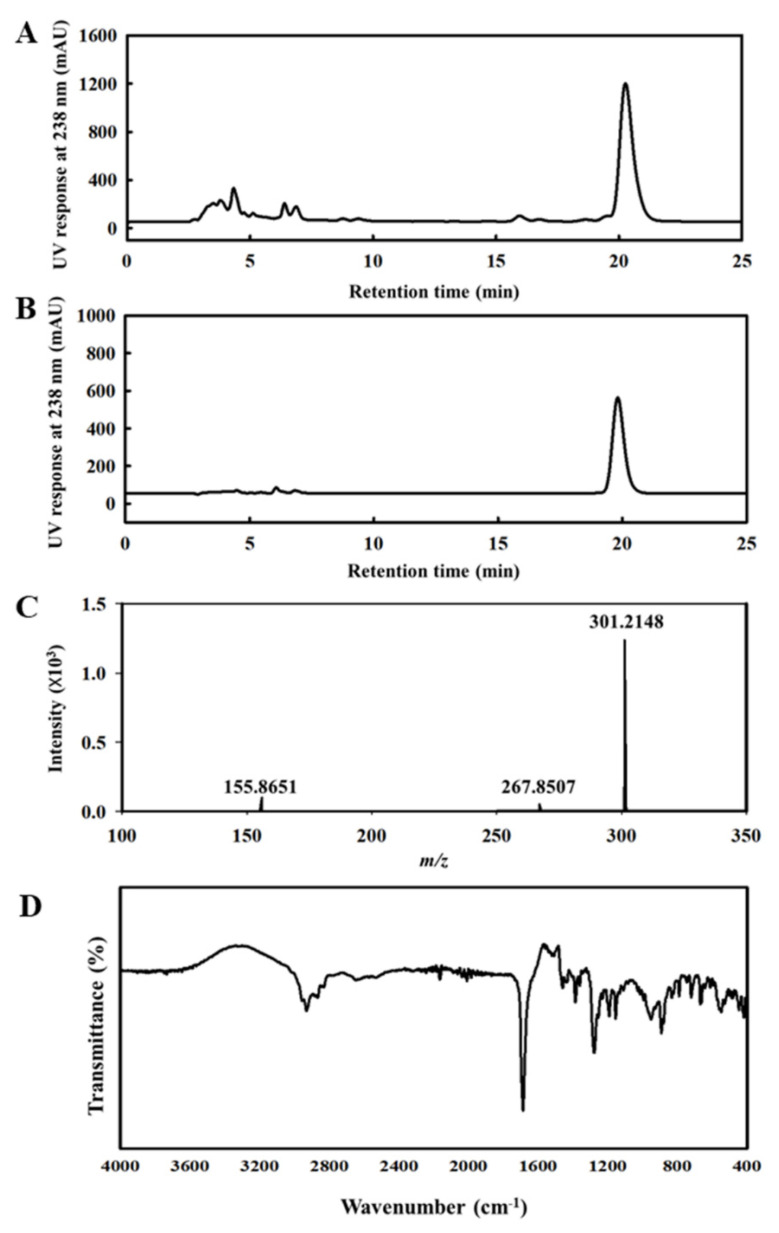
HPLC, LC-MS, and FTIR profile of AA. (**A**) HPLC chromatogram of rosin. (**B**) HPLC chromatogram of AA isolated and purified from rosin. (**C**) LC-MS spectra of AA. (**D**) FTIR spectra of AA. Abbreviations: HPLC, high-performance liquid chromatography; AA, abietic acid; LC-MS, liquid chromatography-mass spectrometry; FTIR, Fourier transform infrared.

To evaluate the purity of AA derived under optimal isolation conditions, HPLC coupled with PDA under the same isocratic conditions was carried out. As shown in Figure 1A,B, AA peaks of AA appeared at 20.3 min and its wavelength spectrum by PDA was identical to the AA standard. The purity of the isolated AA was 99.33%. Furthermore, the MS spectra showed an AA [M-H]- ion at *m/z* 301.2148 as the molecular ion peak (Figure 1C), and its MS spectra showed a fragmentation pattern in good agreement with that of the AA standard.

To further support the assignment of the isolated and purified compound from rosin as AA, the IR spectrum was analyzed (Figure 1D). The IR spectrum of the compound revealed the same patterns as the AA standard, and it showed a large narrow band at 1691 cm^−1^ related to the stretching of C=O, corresponding to the –COOH group. The bands at 2953–2835 cm^−1^ belong to the C–H stretching absorption bands owing to the presence of =CH, –CH_3_, –CH_2_, and –CH groups. In addition, the peaks at 1282 cm^−1^ and 891 cm^−1^ correspond to C–O deformation from the –COOH group and C–H deformation out of the plane of conjugated double bonds, respectively [23,24]. Based on these results, the isolated and purified compound from rosin was identified as AA.

### 2.2. Biochemical Properties of AA against Inflammation and Oxidative Stress

**Figure 2:**
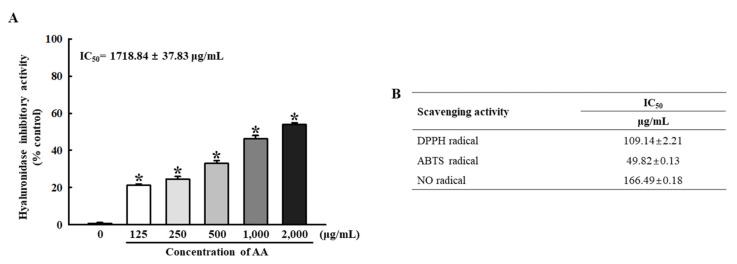
Biochemical properties of AA. (**A**) hyaluronidase HAase activity of AA was calculated by the relative proportion of the sample to the control group after the measurement of optical density at 540 nm. HAase activity analysis was performed with three AA samples in duplicate. (**B**) Radical scavenging activity of AA. These activities against DPPH, ABTS, and NO radicals were measured with a mixture that included appropriate concentrations of radicals and varying concentrations of AA. These levels of AA are represented as IC_50_ values. The scavenging activity of three radicals was analyzed using three AA samples in duplicate. All values in the results are represented as the means ± standard deviation (SD). * indicates statistical significance compared to the non-treated group. Abbreviations: AA, abietic acid; DPPH, 2,2-diphenyl-1-picrylhydrazyl; HAase, hyaluronidase; ABTS, 2,2′-azino-bis (3-ethylbenzothiazoline-6-sulfonic acid); NO, nitric oxide; IC_50_, half the maximum inhibitory concentration.

Next, we analyzed the biochemical properties of AA, newly isolated from rosin, against inflammation and oxidative stress to evaluate its potential for the amelioration of inflammatory diseases. Alterations in hyaluronidase activity and radical scavenging activity were measured at various concentrations of AA. The activity of HAase decreased in a dose-dependent manner with AA. The lowest level (48.31 ± 0.83%) was detected at 2000 μg/mL, followed by 1000 μg/mL (56.40 ± 1.32%), 500 μg/mL (70.32 ± 0.84%), 250 μg/mL (79.09 ± 1.03%), and 125 μg/mL (82.72 ± 1.05%) of AA (Figure 2A). In addition, a similar dose-dependent increase in AA was detected in the AA scavenging activity for the three radicals, including DPPH, ABTS, and NO. The IC_50_ value for the DPPH, ABTS and NO radicals was determined to be 109.14 ± 2.21 µg/mL, 49.82 ± 0.13 µg/mL, and 166.49 ± 0.18 µg/mL (Figure 2B). These results suggest that AA, newly isolated from rosin, shows strong anti-inflammatory and anti-oxidative properties and has the potential to be developed into a therapeutic drug for inflammatory diseases.

### 2.3. Verification of Anti-Inflammatory Activities of AA in LPS-Stimulated RAW264.7 Macrophages

Based on the biochemical properties of AA against inflammation and oxidative stress, we verified the anti-inflammatory effects of AA isolated from rosin in macrophages before evaluating their effects in a DNCB-treated AD model. To achieve this, alterations in cell death, NO production, the iNOS-induced COX-2 mediated pathway, and inflammatory cytokine transcription were analyzed in LPS-stimulated RAW264.7 macrophages.

**Figure 3:**
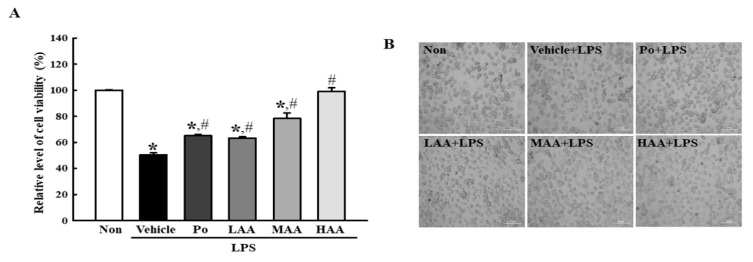
Viability of RAW264.7 cells treated with AA. (**A**) Cell viability. MTT assay was performed on three wells per group and the optical density was analyzed in duplicates. All values in the results are represented as the means ± standard deviation (SD). * indicates statically significance compared to the non-treated group, while # indicates statically significance compared to the Vehicle+LPS-treated group. (**B**) Microscopic image of AA+LPS-treated cells. These cells were observed using a light microscope at 200× magnification. Abbreviations: AA, abietic acid; LPS, lipopolysaccharide; Po, positive control; LAA, low concentration of AA; MAA, medium concentration of AA; HAA, high concentration of AA.

First, changes in cell viability and morphology in RAW264.7 treated with AA+LPS were analyzed to investigate whether treatment with AA newly isolated from rosin can inhibit LPS-induced cell death. Cell viability was 51% lower in the Vehicle+LPS-treated group than in the non-treated group. However, these effects were significantly ameliorated in a dose-dependent manner in the LAA+LPS-, MAA+LPS-, and HAA+LPS-treated groups. In particular, in the HAA+LPS-treated group, these levels were recovered in the non-treated group (Figure 3A). In addition, these alterations in cell viability were fully reflected in the morphology of RAW264.7 macrophages (Figure 3B).

**Figure 4:**
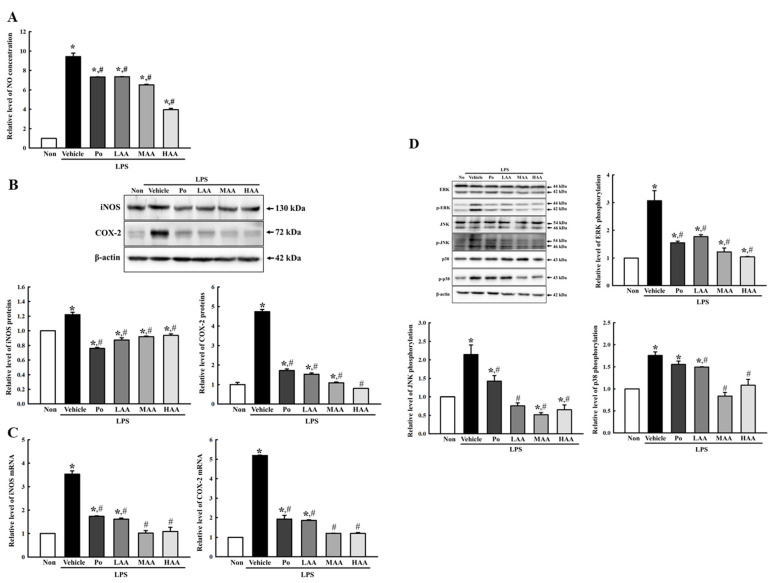
Regulation of the NO-producing system in LPS-stimulated RAW264.7 cells treated with AA. (**A**) NO concentration. NO assay was performed on three wells per group, the optical density was analyzed in duplicate. (**B**) iNOS and COX-2 protein expression. Total cell lysates were added to three to five wells per group and Western blot analyses for each sample were performed in duplicate. (**C**) iNOS and COX-2 mRNA transcription. Total RNA was added to three to five wells per group, and RT-qPCR analyses for each sample were performed in duplicate. (**D**) Expression level of key members of the MAPK signaling pathway. The phosphorylation level of each protein was calculated as a ratio of phosphorylated and unphosphorylated forms. Total cell lysates were added to three to five wells per group, and Western blot analyses were performed for each sample in duplicate. All values in the results are represented as the means ± standard deviation (SD). * indicates statically significance compared to the non-treated group, while # indicates statistical significance compared to the Vehicle+LPS-treated group. Abbreviations: AA, abietic acid; LPS, lipopolysaccharide; Po, positive control; LAA, low concentration of AA; MAA, medium concentration of AA; HAA, high concentration of AA; iNOS, inducible nitric oxide synthase; COX-2, cyclooxygenase-2; ERK, extracellular signal-regulated kinase; JNK, c-Jun N-terminal kinase.

Second, changes in NO concentration and iNOS-induced COX-2 mediated pathway were measured in AA+LPS-treated RAW264.7 macrophages, to investigate whether the treatment of AA newly isolated from rosin can ameliorate against the NO production caused by LPS, The NO concentration was 13.7 times higher in Vehicle+LPS-treated group than in the non-treated group. However, this concentration was markedly ameliorated in a dose-dependent manner after AA treatment (Figure 4A). A similar response was observed in the expression of iNOS and COX-2 proteins in the iNOS- induced COX-2 mediated pathway. The increased iNOS and COX-2 levels in the Vehicle+LPS-treated group were remarkably ameliorated by AA treatment, although a dose-dependent response was observed only in COX-2 expression (Figure 4B). In addition, the expression levels of iNOS and COX-2 proteins were partially reflected in the transcript levels of both genes (Figure 4C). Furthermore, the recovery of iNOS and COX-2 proteins was accompanied by the recovery of the mitogen-activated protein kinase (MAPK) signaling pathway. The enhanced phosphorylation levels of the three key proteins in the Vehicle+LPS-treated group were significantly ameliorated in the three AA+LPS-treated groups (Figure 4D).

**Figure 5:**
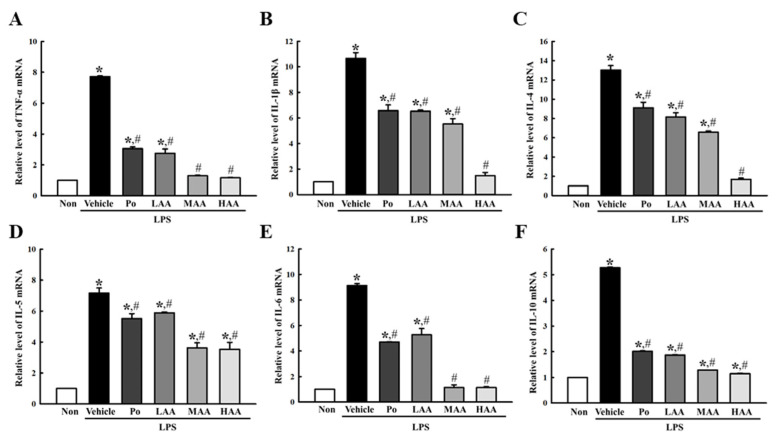
Level of inflammatory cytokines in RAW264.7 cells treated with AA. The levels of (**A**) TNF-α, (**B**) IL-1β, (**C**) IL-4, (**D**) IL-5, (**E**) IL-6, and (**F**) IL-10 transcripts were measured in the total mRNA of AA+LPS-treated RAW264.7 cells by RT-qPCR using specific primers. The mRNA levels of the six genes were calculated based on the intensity of β-actin as an endogenous control. The total RNAs were added to three to five wells per group, and RT-qPCR analyses for each sample were analyzed in duplicate. All values in the results are represented as the means ± standard deviation (SD). * indicates statically significance compared to the non-treated group, while # indicates statically significance compared to the Vehicle+LPS-treated group. Abbreviations: AA, abietic acid; LPS, lipopolysaccharide; Po, positive control; LAA, low concentration of AA; MAA, medium concentration of AA; HAA, high concentration of AA; TNF-α, tumor necrosis factor alpha; IL, interleukin.

Third, the transcription levels of inflammatory cytokines were measured in the AA+LPS-treated RAW264.7 macrophages, to examine whether the amelioration effects of AA newly isolated from rosin on LPS-induced NO production are accompanied by alterations in cytokine secretions. The regulation of inflammatory cytokine transcription is well reflected in the regulation of NO production and the iNOS-induced COX-2-mediated pathway. The AA+LPS-treated groups exhibited a significant amelioration in the transcript levels of six cytokines, including TNF-α, IL-1β, IL-4, IL-5, IL-6, and IL-10, compared to the Vehicle+LPS-treated group. Most of them showed the highest decrease in the HAA+LPS-treated group, although a dose-dependent response was detected in the expression of IL-4 (Figure 5).

**Figure 6:**
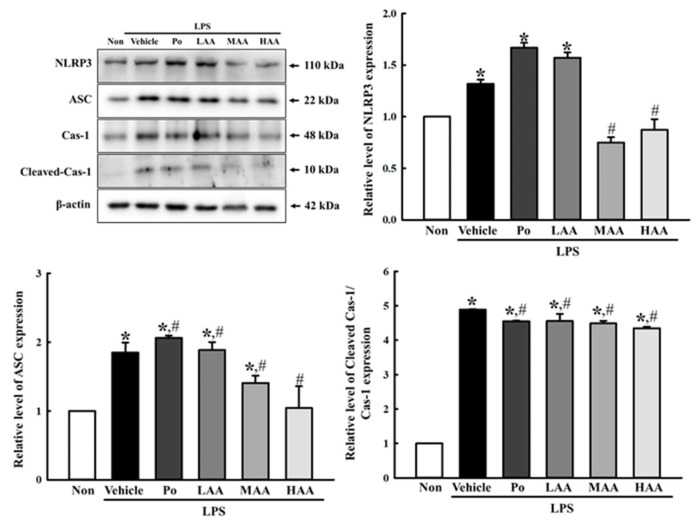
Inflammasome levels in RAW264.7 cells treated with AA. After total cell lysates were prepared from AA+LPS-treated RAW264.7 cells, the expression levels of the key regulators including NLRP3, ASC, Cas-1, Cleaved Cas-1, and β-actin were detected using Western blot analysis. Total cell lysates were prepared from three to five wells per group, and Western blot analyses for each sample were analyzed in duplicate. All values in the results are represented as the means ± standard deviation (SD). * indicates statically significance compared to the non-treated group, while # indicates statistical significance compared to the Vehicle+LPS-treated group. Abbreviations: AA, abietic acid; LPS, lipopolysaccharide; Po, positive control; LAA, low concentration of AA; MAA, medium concentration of AA; HAA, high concentration of AA.

Finally, a similar amelioration effect of AA in the NO-producing system was reflected in inflammasome activation. The increased levels of NLR family pyrin domain containing 3 (NLRP3), apoptosis-associated speck-like protein containing a CARD (ASC), and the cleavage of Cas-1 proteins in the Vehicle-treated group were significantly decreased in the LAA+LPS-, MAA+LPS-, and HAA+LPS-treated groups compared to those in the Vehicle+LPS-treated group (Figure 6). Therefore, the results of all experiments indicate that AA, newly isolated from rosin, may contribute to the amelioration of the increase in cell death, NO production systems, and inflammatory cytokine transcription in LPS-stimulated RAW264.7 macrophages.

### 2.4. Amelioration of Skin Phenotypes in DNCB-Induced AD Mice by AA Spreading

**Figure 7:**
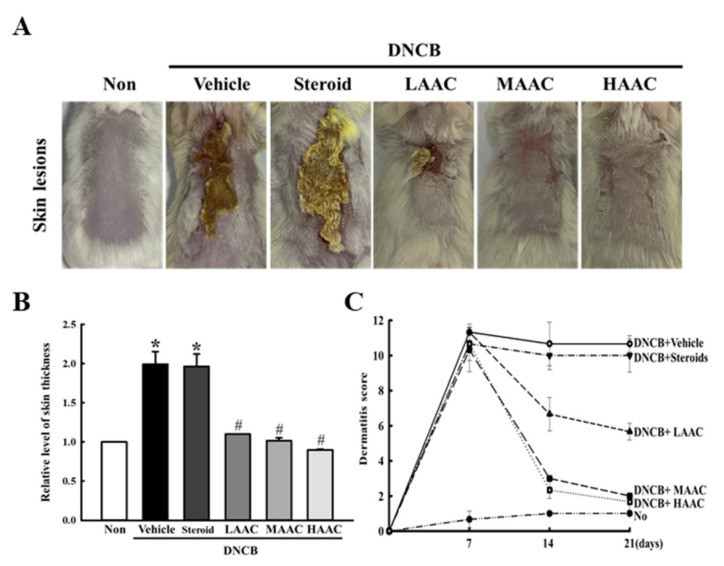
Skin phenotypes of DNCB-treated BALB/c mice spread with AAC. (**A**) Actual image of skin lesions. After the final treatment, images were taken with a digital camera. (**B**) Level of skin thickness. Before a necropsy, the thickness of the skin in mice of subset groups was measured using a thickness gauge, as described in the Materials and Methods. (**C**) Dermatitis score. During the experimental period, the dermatitis scores were determined based on the SCORing Atopic Dermatitis (SCORAD) index. The skin lesion and thickness experiments were performed on four to six mice per group, and the parameters for these experiments were analyzed in duplicate. All values in the results are represented as the means ± standard deviation (SD). * indicates statically significance compared to the non-treated group, while # indicates statically significance compared to the DNCB+Vehicle-treated group. Abbreviations: AA, abietic acid; DNCB, 2,4-dinitrochlorobenzene; LAAC, low concentration of AA cream; MAAC, medium concentration of AA cream; HAAC, high concentration of AA cream.

Furthermore, we investigated the ameliorative effects of AA, newly isolated from rosin, against AD symptoms in DNCB-treated BALB/c mice to confirm the same effects of AA detected in LPS-stimulated RAW264.7 macrophages. As part of these experiments, alterations in skin thickness and dermatitis score were first analyzed in the skin of the DNCB-induced AD model after AA spreading. As shown in Figure 7A,B, skin thickness was greater in the DNCB+Vehicle-treated group than in the non-treated group. However, this level was markedly ameliorated in a dose-dependent manner after AA spread, although the DNCB+Steroid-treated group remained constant. In addition, a similar response to AA was observed in the dermatitis score. After treatment with AA for 14 days, these scores were significantly ameliorated in the DNCB+LAAC-, DNCB+MAAC-, and DNCB+HAAC-treated groups compared to the DNCB+Vehicle-treated group, although the highest decrease rate was detected in the DNCB+HAAC-treated group (Figure 7A,C). Therefore, these results indicate that the spread of AA newly isolated from rosin can ameliorate the deterioration of skin phenotypes in a DNCB-induced AD model.

### 2.5. Amelioration of IgE-Mediated Symptoms in DNCB-Induced AD Mice by AA Spreading

**Figure 8:**
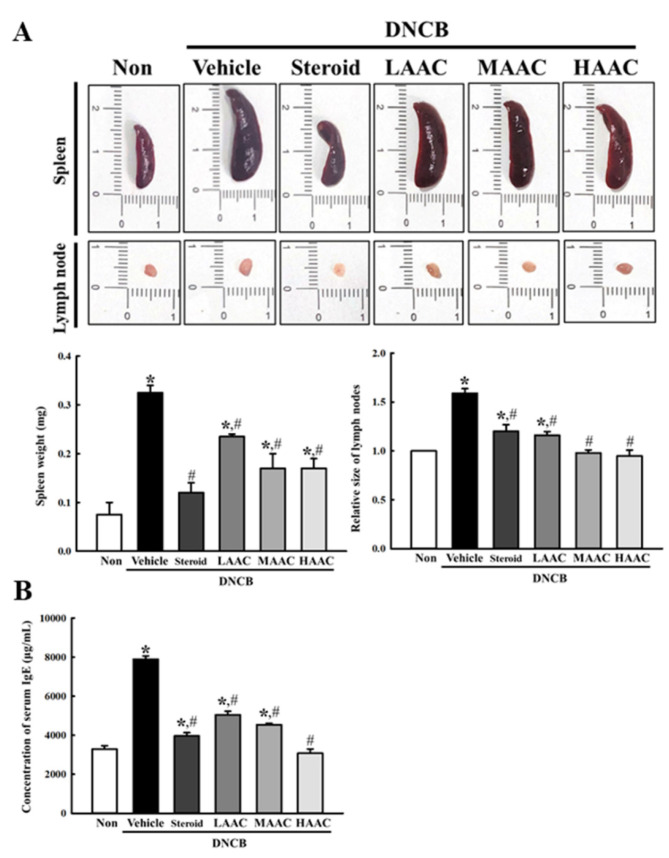
Alterations of IgE-mediated symptoms in DNCB-treated BALB/c mice spread with AAC. (**A**) Weight of immune organs. After the collection of the spleen and lymph node, their actual images were taken with a digital camera, and their weights were measured using an electrical balance. (**B**) The concentration of IgE was quantified in serum using an enzyme-linked immunosorbent assay (ELISA) with a detectable concentration range of 10–5000 ng/mL. Organs and serum were collected from four to six mice per group, and the weight of organs and IgE concentrations were analyzed in duplicate. All values in the results are represented as the means ± standard deviation (SD). * indicates statically significance compared to the non-treated group, while # indicates statistical significance compared to the DNCB+Vehicle-treated group. Abbreviations: AA, abietic acid; DNCB, 2,4-dinitrochlorobenzene; LAAC, low concentration of AA cream; MAAC, medium concentration of AA cream; HAAC, high concentration of AA cream; IgE, immunoglobulin E.

To determine whether the ameliorative effects of AA on DNCB-induced deterioration of the skin phenotype are accompanied by alterations in IgE-mediated symptoms, the weight of the immune organ and IgE concentration were measured in the DNCB-induced AD model after spreading AA. First, the weight of the spleen and the relative size of the lymph node were remarkably higher in the DNCB+Vehicle-treated group than in the non-treated group. However, these alterations were ameliorated in the DNCB+AAC-treated groups compared to the DNCB+Vehicle-treated group (Figure 8A). A similar response was observed for the concentration of serum IgE. The increased level in the DNCB+Vehicle-treated group was ameliorated in a dose-dependent manner in the DNCB+AAC group (Figure 8B). Thus, the above results indicate that the ameliorative effects of AA on DNCB-induced deterioration of the skin phenotype may be tightly linked to the alleviation of IgE-mediated symptoms in the DNCB-induced AD model.

### 2.6. Amelioration of Skin Histopathological Structure in DNCB-Induced AD Mice by AA Spreading

**Figure 9:**
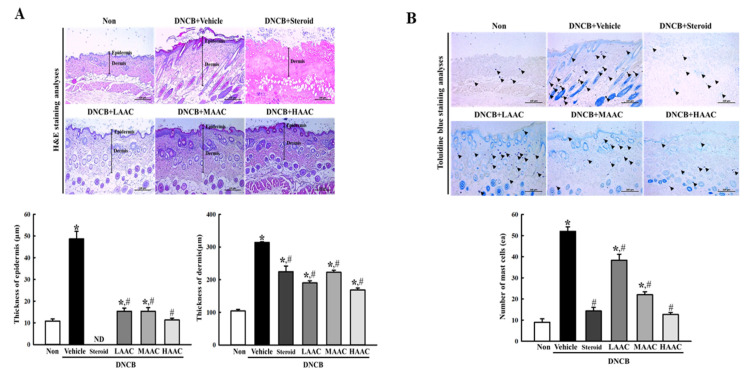
Histopathological structure of the skin in DNCB-treated BALB/c mice spread with AAC. (**A**) Alteration of skin histological structure. After staining with H&E solution, their structures were observed at 400× magnification using an optical microscope. The thickness of the epidermis and dermis was measured by Image J program. (**B**) Infiltration of mast cells. After staining with TB, the number of mast cells was calculated by observation at 400× magnification. Arrowheads indicate the infiltrated mast cells in the skin tissue. The H&E and TB-stained slides were prepared from four to six mice per group, and the histopathological parameters were analyzed in duplicate. All values in the results are represented as the means ± standard deviation (SD). * indicates statically significance compared to the non-treated group, while # indicates statistical significance compared to the DNCB+Vehicle-treated group. Abbreviations: AA, abietic acid; DNCB, 2,4-dinitrochlorobenzene; LAAC, low concentration of AA cream; MAAC, medium concentration of AA cream; HAAC, high concentration of AA cream; H&E, hematoxylin and eosin; TB, toluidine blue; ND, not detected.

To determine whether the ameliorative effects of AA on DNCB-induced deterioration of skin phenotype were accompanied by alterations in the histopathological structure of the skin, the thickness of the dermis and epidermis and the number of mast cells were measured in the skin of the DNCB-induced AD model after AA spreading. The thickness of the epidermis and dermis was higher in the DNCB+Vehicle-treated group than in the non-treated group. However, these levels were significantly ameliorated after AAC treatment when compared to those in the DNCB+Vehicle-treated group (Figure 9A). Additionally, the ameliorative effects of AAC on the thickness of the epidermis and dermis were completely reflected by the infiltration of immune cells.

The increased number of mast cells was ameliorated in the DNCB+LAAC-, DNCB+MAAC-, and DNCB+HAAC-treated groups compared to that in the DNCB+Vehicle-treated group (Figure 9B). Therefore, these results suggest that the ameliorative effects of AA on DNCB-induced deterioration of the skin phenotype may be closely related to the alleviation of the histopathological structure of the skin in the DNCB-induced AD model.

### 2.7. Amelioration of NO-Producing System in DNCB-Induced AD Mice by AA Spreading

**Figure 10:**
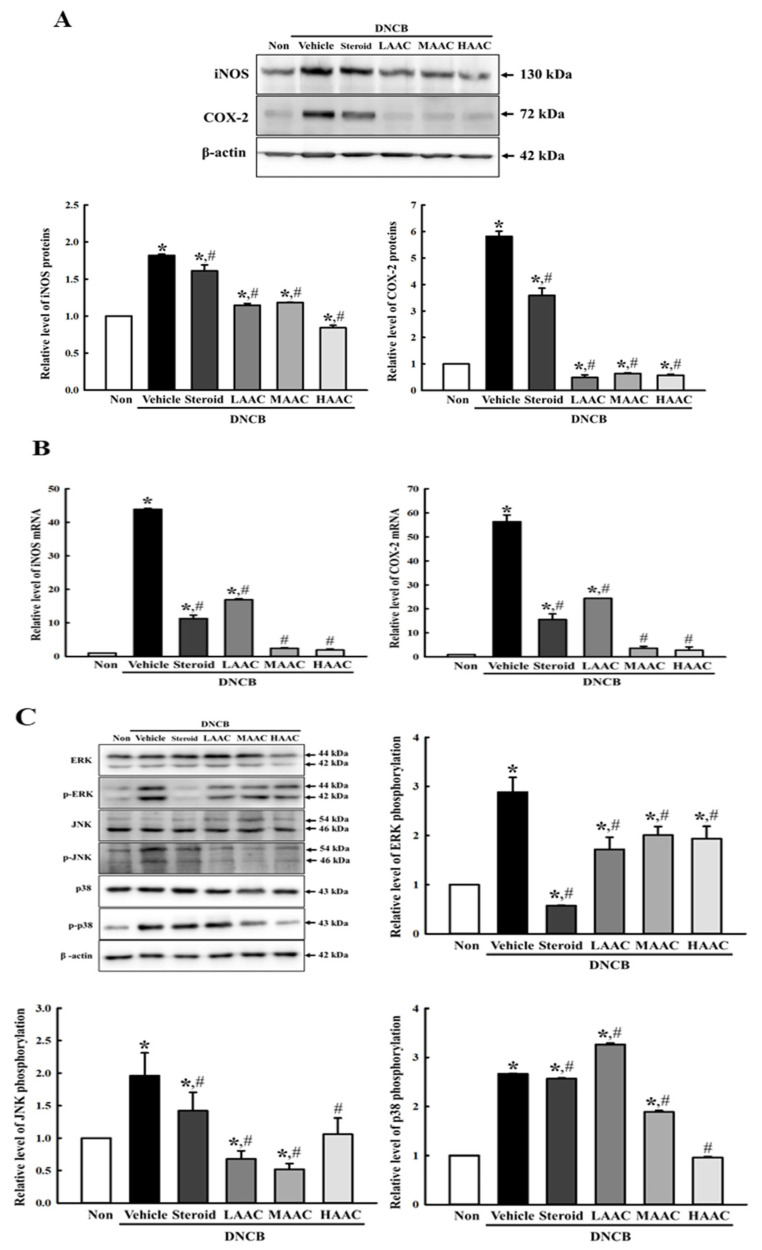
Regulation on NO producing system in DNCB-treated BALB/c mice spread with AAC. (**A**) iNOS and COX-2 protein expression. After the preparation of total tissue homogenate from DNCB+AAC-treated BALB/c mice, the expression levels of the two proteins were detected using Western blot. (**B**) iNOS and COX-2 mRNA transcription. The levels of iNOS and COX-2 transcripts were measured in the total mRNA of DNCB+AAC-treated BALB/c mice by RT-qPCR using specific primers. (**C**) Expression levels of key members in MAPK signaling pathway. The phosphorylation level of each protein was calculated as the ratio of phosphorylated to unphosphorylated forms. The total proteins homogenates and RNAs were prepared from four to six mice per group, and Western blot and RT-qPCR analyses for each sample were performed in duplicate. All values in the results are represented as the means ± standard deviation (SD). * indicates statically significance compared to the non-treated group, while # indicates statistical significance compared to the DNCB+Vehicle-treated group. Abbreviations: AA, abietic acid; DNCB, 2,4-dinitrochlorobenzene; LAAC, low concentration of AA cream; MAAC, medium concentration of AA cream; HAAC, high concentration of AA cream; iNOS, inducible nitric oxide synthase; COX-2, cyclooxygenase-2; ERK, extracellular signal-regulated kinase; JNK, c-Jun N-terminal kinase.

To determine whether the ameliorative effects of AA on DNCB-induced deterioration of the skin phenotype are accompanied by alterations in the NO production system, the expression levels of iNOS, COX-2, and MAPK members were measured in the skin of the DNCB-induced AD model after AA spreading. First, the expression levels of iNOS and COX-2 proteins were increased in the DNCB+Vehicle-treated group compared to the non-treated group. However, these levels were remarkably ameliorated in the DNCB+LAAC-, DNCB+MAAC-, and DNCB+HAAC-treated groups (Figure 10A). In addition, these alteration patterns in the protein levels of iNOS and COX-2 were detected at the mRNA levels of both genes (Figure 10B). Furthermore, the amelioration of the expression levels of iNOS and COX-2 proteins was accompanied by the recovery of the activation of the MAPK signaling pathway. The increased levels of ERK, JNK, and p38 phosphorylation were remarkably ameliorated in the DNCB+AAC-treated groups compared to those in the DNCB+Vehicle-treated group (Figure 10C). Thus, these results indicate that the ameliorative effects of AA on DNCB-induced deterioration of the skin phenotype may be closely related to the alleviation of the NO production system by regulating the iNOS-mediated COX-2 induction pathway, including the MAPK signaling pathway.

### 2.8. Amelioration of Inflammatory Cytokines Secretion in DNCB-Induced AD Mice by AA Spreading

**Figure 11:**
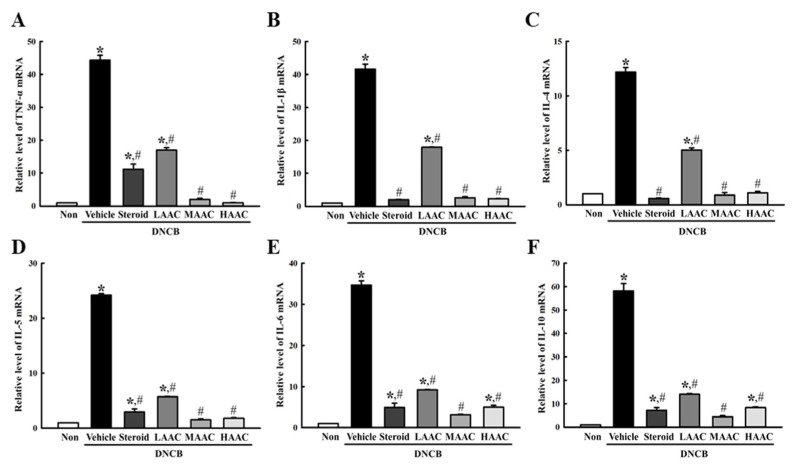
Level of inflammatory cytokines in DNCB-treated BALB/c mice spread with AAC. The levels of (**A**) TNF-α, (**B**) IL-1β, (**C**) IL-4, (**D**) IL-5, (**E**) IL-6, and (**F**) IL-10 transcripts were measured in the total mRNA of DNCB+AAC-treated BALB/c mice by RT-qPCR using specific primers. The mRNA levels of the six genes were calculated based on the intensity of actin as an endogenous control. Total RNA was prepared from four to six mice per group, and RT-qPCR analyses for each sample were analyzed in duplicate. All values in the results are represented as the means ± standard deviation (SD). * indicates statically significance compared to the non-treated group, while # indicates statistical significance compared to the DNCB+Vehicle-treated group. Abbreviations: AA, abietic acid; DNCB, 2,4-dinitrochlorobenzene; LAAC, low concentration of AA cream; MAAC, medium concentration of AA cream; HAAC, high concentration of AA cream; TNF-α, tumor necrosis factor alpha; IL, interleukin.

To examine whether the ameliorative effects of AA on DNCB-induced deterioration of skin phenotype were accompanied by alleviation of inflammatory cytokine secretion, the expression levels of six cytokines, including TNF-α, IL-1β, IL-4, IL-5, IL-6, and IL-10, were measured in the skin tissues of DNCB+AAC-treated mice. Most of them showed similar response patterns after AAC spreading. The mRNA levels of six cytokines were higher in the DNCB+Vehicle-treated group than in the non-treated group. However, they were significantly ameliorated in the DNCB+AAC group compared to the DNCB+Vehicle-treated group, although dose-dependent responses were not clearly observed (Figure 11). Therefore, these results suggest that the ameliorative effects of AA on DNCB-induced deterioration of the skin phenotype may be related to the alleviation of inflammatory cytokine transcription.

### 2.9. Amelioration of Inflammasome Activation in DNCB-Induced AD Mice by AA Spreading

**Figure 12:**
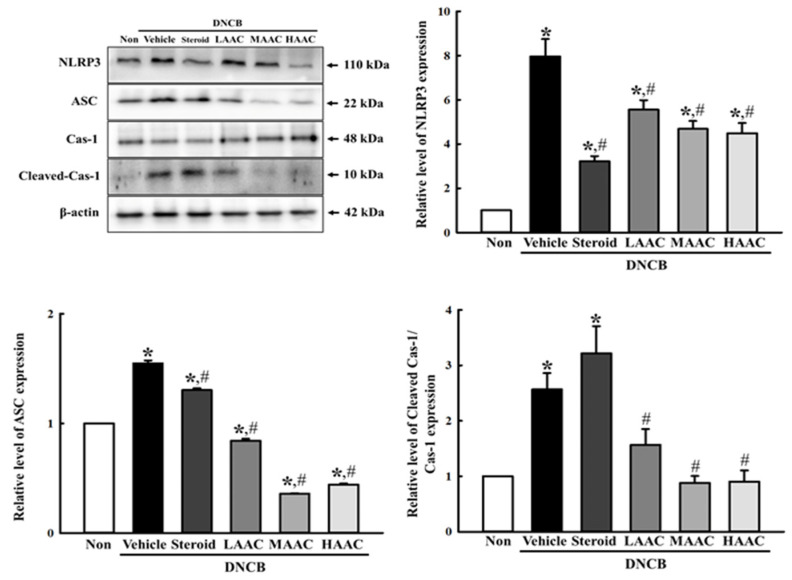
Inflammasomes levels in DNCB-treated BALB/c mice spread with AAC. After preparation of tissue homogenate from DNCB+AAC-treated BALB/c mice, the expression levels of the key regulators including NLRP3, ASC, Cas-1, Cleaved Cas-1, and β-actin were detected using Western blot analysis. The total proteins homogenates were prepared from five to six mice per group, and Western blot analyses for each sample were analyzed in duplicate. All values in the results are represented as the means ± standard deviation (SD). * indicates statically significance compared to the non-treated group, while # indicates statistical significance compared to the DNCB+Vehicle-treated group. Abbreviations: AA, abietic acid; DNCB, 2,4-dinitrochlorobenzene; LAAC, low concentration of AA cream; MAAC, medium concentration of AA cream; HAAC, high concentration of AA cream.

Finally, we investigated whether the ameliorative effects of AA on DNCB-induced deterioration of the skin phenotype are accompanied by the alleviation of inflammasome activation. To achieve this, the expression levels of key regulators, including NLRP3, ASC, cleaved Cas-1, and Cas-1, were analyzed in the skin tissue of DNCB+AAC-treated mice. Their levels were higher in the DNCB+Vehicle-treated group than in the non-treated group. However, these levels were remarkably decreased in all DNCB-treated groups after spreading AAC for 28 days (Figure 12). Therefore, the above results indicate that the ameliorative effects of AA on DNCB-induced deterioration of the skin phenotype may be associated with the alleviation of inflammasome activation.

## 3. Discussion

Inflammatory responses stimulated by pathogens, toxic compounds, and damaged cells can remove harmful factors and initiate the healing mechanism and lead to tissue damage in various organs, including the brain, heart, lung, and liver [25]. Therefore, mitigation of this response can be considered an effective strategy to recover tissue homeostasis and ameliorate inflammatory diseases [26]. As part of these studies, a treatment strategy using AA has recently received considerable attention because it is being studied for its therapeutic efficacy and mechanism of action in several inflammation-related diseases, such as inflammatory bowel disease, rheumatoid arthritis, and psoriasis [27]. In this study, the optimal isolation condition including amount of HCl (2.49 mL), reflux extraction time (61.7 min), and amount of ethanolamine (7.35 mL) was firstly established using RSM. Under these conditions, the purity and yield of AA were enhanced to 99.33% and 58.61%. Additionally, anti-AD effects and related mechanisms of AA were investigated in a DNCB-treated mouse model. Our results provide novel scientific evidence that AA with high purity was isolated in high yield under RSM-optimized conditions, and that this AA may contribute to the amelioration of DNCB-stimulated AD phenotypes through the regulation of the iNOS-induced COX-2 mediated pathway and inflammatory cytokine production.

Rosin is an important non-timber forest material that is naturally derived from the oozed colophony of pine trees [28,29]. The main source of abietane acid is rosin (colophony), which is the residue of the distillation of pine resins. The acid part of rosin is composed of AA, its equilibrium isomers such as levopimaric, palustric, and neoabietic acids, and dehydroabietic acid, as well as some other non-abietanic compounds [30,31,32,33]. The main ingredients of rosin are terpene-based neutral compounds (10–20%) and abietic-type resin acids (80–90%), with the main component (approximately 50%) being AA [34,35]. A study proposed the process of isolating AA from rosin via isomerization, amination, and crystallization [11]. They obtained AA with a purity and yield of 98.52% and 54.93%, respectively. However, the recrystallization of the amine salts was repeated three times. In this study, the isomerization and reaction-crystallization for AA was optimized with a certain amount of HCl (2.49 mL), reflux extraction time (61.7 min), and ethanolamine (7.35 mL) using RSM. After the crystallization of AA, preparative HPLC was performed. Finally, the purity and yield of AA were improved to 99.33% and 58.61%, respectively, although the AA isolation process was simplified.

HAase is a hyaluronic acid (HA) hydrolyzing enzyme that is related to physiological regulatory processes and pathological conditions, including inflammation and allergic responses [36,37]. The purified AA inhibited HAase activity in a dose-dependent manner with an IC_50_ value of 1719 μg/mL (Figure 2A). During inflammation and tissue damage, HAase cleaves HA into fragments of lower molecular weight, thus inducing pro-inflammatory immune responses [38]. It has been reported that AA from *Pimenta racemose* var. *grissea* exerted anti-inflammatory activity against edema and had a partial ability to prevent the production of some inflammatory mediators [39]. Therefore, the inhibitory activity of AA on HAase may play an important role in the pathological processes of AD pathogenesis.

The anti-oxidant activities of AA were evaluated by measuring its radical scavenging effects on DPPH, ABTS, and NO radicals. The concentrations required to scavenge 50% of the initial DPPH, ABTS, and NO radicals were 109.14 μg/mL, 49.82 μg/mL, and 166.49 μg/mL, respectively. In addition, a study reported that IC_50_ values of DPPH and hydroxyl radicals toward AA of *Isodon wightii* were calculated as 660.36 μg/mL and 467.43 μg/mL [40]. The anti-oxidant activities of other abietane diterpenoids, such as carnosol, isorosmanol, carnosic acid, rosmanol, epirosmanol, and galdosol from *Salvia officinalis* L. and inuroyleanol from *S. barrelieri*, have been reported in the literature [41,42]. It has been suggested that the anti-oxidant mechanism of abietane diterpenoids in nonpolar environments involves formal hydrogen transfer, whereas the single-electron transfer mechanism of anion states in aqueous environments is favored [43]. These results indicate that AA serves as an effective free radical scavenger.

LPS is well known as a potential activator of monocytes and macrophages because it induces an acute inflammatory response by stimulating the secretion of a vast number of inflammatory cytokines in many types of cells [44,45]. Based on these properties, LPS-stimulated cell models have been widely used to evaluate the anti-inflammatory effects of AA or natural products containing AA. First, AA isolated from *Pimenta racemosa* var. *grisea* (Myrtaceae) significantly prevented the production of NO, Prostaglandin E2 (PGE2), TNF-α, and IL-1β in LPS-stimulated peritoneal macrophages after treatment with 10 and 100 μM concentrations [39]. In addition, the protein expression of TNF-α and COX-2 through the regulation of peroxisome proliferator-activated receptor gamma (PPARγ) in LPS-stimulated peritoneal macrophages was suppressed by 25 and 50 µM of AA and induced similar effects on the production of inflammatory mediators including IL-1β, IL-6, Macrophage inflammatory protein (MIP)-2, and TNF-α in LPS-stimulated RAW264.7 cells after treatment with 20, 40, and 80 µmol/L of AA [16,46]. The enhancement of NO, PGE2, iNOS, COX-2, IL-1β, IL-6, and TNF-α production stimulated by LPS was inhibited by low concentrations of AA (5, 10, 20, and 40 µmol/L) in LPS-stimulated RAW264.7 cells [47]. In this study, we evaluated the potential anti-inflammatory effects of AA on AD symptoms. The levels of various inflammatory mediators were remarkably ameliorated by 10, 20, and 40 µM AA. These results are similar to those of previous studies that examined the inhibition of inflammatory mediators in LPS-stimulated macrophages after treatment with different concentrations of AA derived from different sources. Furthermore, our results suggest that AA isolated from rosin under conditions optimized by RSM has similar effects on the inflammatory response to those isolated by previously reported methods [39,46,47].

The therapeutic efficacy of AA has been studied in several animal models of inflammatory disease. The anti-allergic effects of AA were investigated in ovalbumin (OVA)-induced asthmatic mice after treatment with 10, 20, and 40 mg/kg. OVA-induced airway hyper-responsiveness, inflammatory cell infiltration, NO production, OVA-specific IgE production, cytokine secretion, and NF-κB activation were attenuated by treatment with AA isolated from *P. racemosa* var. *grisea* [22]. In addition, the wound healing effects of AA isolated from pine resin (Resina Pini) were analyzed in an ICR model with a 5 mm full-thickness excisional skin wound after treatment with 0.8 µM for 10 days based on angiogenic potential, including cell migration, tube formation, and activation of ERK/p38 in human umbilical vein vascular endothelial cells (HUVECs). Wound closure was accelerated in the AA-treated group compared with that in the control group [15]. A similar improvement effect of AA was detected in IMQ-induced psoriasis-like inflammation in a BALB/c mouse model after treatment with 40 mg/kg/day AA for 7 days. Sodium abietate decreased Psoriasis Area Severity Index (PASI) scores, ameliorated the balance of Th17/Treg cells in the spleen, decreased inflammatory cytokine secretion, and recovered the alteration of gut microbiota [20]. In this study, we investigated the mitigating effects and action mechanisms of AA, newly isolated from rosin, in an AD model as part of a study to identify novel functions of AA. Significant amelioration effects on skin thickness, dermatitis score, immune organ weight, IgE concentration, the histopathological structure of the skin, iNOS-induced COX-2 mediated pathway, and secretion of inflammatory cytokines were detected in DNCB-spread BALB/c mice after treatment with AAC for 28 days. Therefore, these results in DNCB-spread BALB/c models suggest a novel therapeutic role for AA, which has not been previously investigated. Furthermore, the results of the present study suggest that AA can successfully ameliorate inflammatory diseases.

## 4. Materials and Methods

### 4.1. RSM and Central Composite Design (CCD) Analysis

RSM and CCD analysis was employed to maximize the extraction yield and improve the purity of AA from rosin. The experimental design and data analysis were performed using with a design Expert 8 program (State-Easy Co., Minneapolis, MN, USA). The ranges and coded levels of the process variables were the amount of HCl added (X_1_, 0.5–3.0 mL), reflux extraction time (X_2_, 20–90 min), and amount of ethanolamine added (X_3_, 0–20 mL). The dependent values were the extraction yield and purity of AA. The RSM design consisted of 17 combinations and the levels of each factor are listed in Supplementary Table S1. During optimization of the extraction yield and the purity of AA, the response could be related to chosen factors by the full quadratic model. The analysis of variance (ANOVA) was used to evaluate the significance and regression analysis was performed on the data of response variables. The quality of fit the polynomial model was expressed by the regression coefficients (R2), and its statistical significance was checked by an F test in the same program.

### 4.2. Isolation and Purification of AA from Rosin

**Figure 13:**
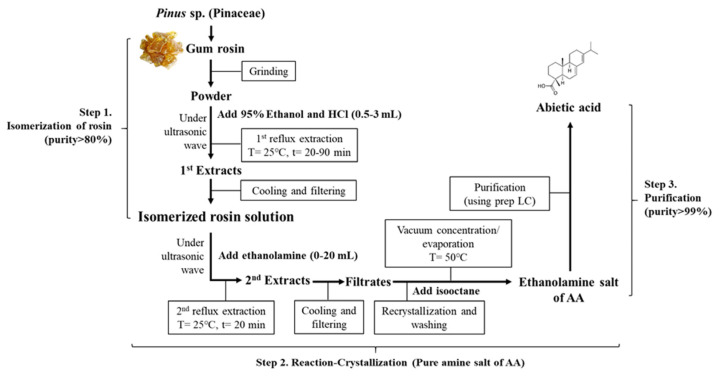
Experimental scheme for isolation, crystallization and purification of AA from rosin.

Rosin from *Pinus merkusii* was purchased from Resin Chemicals Co., Ltd. (Guangzhou, China). Freeze-dried rosin was powdered to a size of 100 µm. AA was isolated and purified under the condition optimized by RSM and CCD analysis based on the previous method [11]. Briefly, AA was purified through a series of isomerization, reaction-crystallization, and purification step (Figure 13). The amine salt of AA obtained under optimal conditions was further purified using preparative high performance liquid chromatography (HPLC) (LC-forte/R, YMC Co., Kyoto, Japan). This was carried out under isocratic conditions with 0.05% formic acid in water/methanol using a YMC Triart Prep C18-S column (250 × 10.0 mm, 10 μm; YMC Co.). Purified AA was used in the present study. Voucher specimens of dried AA (WPC-22-001) were deposited at the Functional Materials Bank (FMB) of the Pusan National University (PNU)–Wellbeing RIS Center.

### 4.3. HPLC, LC-MS, and FTIR Analysis

HPLC was performed to evaluate the purity of AA isolated from rosin. A HPLC system (iLC3000, Interface Engineering Co., Ltd., Seoul, Republic of Korea) with a photodiode array detector (PDA; S3210, BMS Co., Ltd., Seoul, Republic of Korea) was used. Chromatographic separation was carried out using a YMC-Triart C18 column (4.6 × 250 mm, 5 µm; YMC Co.). The mobile phase consisted of 0.1% formic acid in water (Solvent A) and methanol (Solvent B). The mobile phase was methanol/water/formic acid (87/12.95/0.05), the flow rate was 0.8 mL/min, and 20 µL of the sample was injected. Compounds separated by chromatography were detected using PDA. Data acquisition and preprocessing were performed using the Clarity™ chromatography software (DataApex, Prague, Czech Republic).

Liquid chromatography was carried out using an ACQUITY UPLC BEH C18 (2.1 × 100 mm, 1.7 μm) column (Waters, Milford, MA, USA). Mass spectrometric detection was performed using an Agilent 1290 Infinity HPLC system (Agilent Technologies, Waldbronn, Germany) coupled with a hybrid quadrupole time-of-flight (Q-TOF) mass spectrometer (TripleTOF 4600; AB Sciex Pte. Ltd., Framingham, MA, USA). The source parameters were as follows: gas temperature, 300 °C; gas flow, 9 L/min; nebulizer pressure, 45 psig; sheath gas temperature, 350 °C; sheath gas flow, 11 L/min; and negative mode ([M–H]^−^ ions). The scan source parameters were a VCap 4000 V and a fragmentor voltage of 175 V.

Attenuated total reflection-Fourier transform infrared (ATR-FTIR) spectra were measured on an FTIR spectrometer (Nicolet iS50, Thermo Fisher Scientific Inc., Waltham, MA, USA) interfaced with an ATR device on its Monolithic Diamond ATR Crystal. Spectra were recorded in the mid-infrared (MIR) region between 4000 and 400 cm^−1^ (2.5–25 μm). The data obtained data were analyzed using OMNIC™ FTIR Software (Thermo Fisher Scientific Inc.). The background spectrum of the air was recorded under the same instrumental conditions before each sample was read.

### 4.4. Assay for Hyaluronidase (HAase) Inhibition Activity

HAase inhibition activity was measured using the colorimetric Morgan–Elson assay with slight modification to measure the quantity of *N*-acetylglucosamine generated from sodium hyaluronate [48]. Each sample dissolved in dimethyl sulfoxide (DMSO; Duchefa Biochemie, Haarlem, the Netherlands) (100 mg/mL) was diluted with 0.1 M sodium acetate buffer (pH 3.5) for the test. A 12 µL sample diluted with 0.1 M sodium acetate and 12 µL of HAase (10 mg/mL) was mixed and incubated in a water bath at 37 °C for 20 min. To activate HAase, 12 µL of 12.5 mM calcium chloride was added and incubated at 37 °C for 20 min. Subsequently, 24 µL of sodium hyaluronate (6 mg/mL) was reacted with the sample mixture at 37 °C for 40 min. Both HAase and sodium hyaluronate are soluble in 0.1 M sodium acetate buffer. A total of 0.4 N potassium tetraborate and 0.4 N NaOH (12 µL) were added to quench the HAase reactions.

To completely terminate HAase activity, the samples were placed in boiling water for 3 min and then on ice. Finally, 360 µL of *p*-dimethylaminobenzaldehyde (DMAB) solution (0.4 g of DMAB reagent in 35 mL of glacial acetic acid and 5 mL of 10 N HCl) was added to the reaction mixture with a vortex and incubated in an incubation water bath at 37 °C for 20 min. All tubes in the test were measured using a microplate reader (Tecan Sunrise, Tecan, Hombrechtikon, Switzerland) at 540 nm after centrifugation for a few seconds. The percentage of HAase activity was determined using the following equation:

HAase activity (%) = (Abs_sample_/Abs_control_) × 100

### 4.5. Free Radical Scavenging Activity Assay

The scavenging activity of AA against 1,1-diphenyl-2-picrylhydrazyl (DPPH) radicals was measured using a previously reported method [49,50]. After preparing DPPH solutions of different concentrations, 100 μL of the AA solution was mixed with the same volume of the DPPH solution, and this mixture was incubated at room temperature for 30 min in the dark. The absorbance of each mixture was measured at 540 nm wavelength using a VersaMax^TM^ plate reader (Molecular Devices, Sunnyvale, CA, USA). The scavenging activity of AA against DPPH radicals was represented as the half-maximal inhibitory concentration (IC_50_) value, which is defined as the concentration of AA inducing a 50% loss in DPPH radical scavenging activity.

The scavenging activity of AA against 2,2′-azino-bis (3-ethylbenzthiazoline-6-sulfonic acid) (ABTS) radicals was determined as previously reported [51]. A total of 25 μL of 11 different concentrations of AA (1–500 µg/mL) was mixed with 250 μL of ABTS working solution and incubated at room temperature for 4 min. The absorbance of the reaction mixture was read at 734 nm using a UV-visible (UV–VIS) spectrophotometer (Thermo Fisher Scientific Inc.). Finally, the ABTS radical scavenging activity of AA was represented as the half-maximal IC_50_ value.

The scavenging activity of AA against nitric oxide (NO) radicals was determined as reported in a previous study using the modified Jaiswal method [52,53]. Briefly, 100 µL of AA solution was mixed with 400 µL of 10 mM sodium nitroprusside and incubated at room temperature for 2.5 h. This mixture was reacted with 200 µL of Griess reagent for 30 min. Absorbance was measured at 540 nm using a VersaMax^TM^ plate reader (Molecular Devices). The scavenging activity of AA against NO radicals was represented as the half-maximal IC_50_ value.

### 4.6. Cell Culture and Viability

RAW264.7, a macrophage cell line from tumor in male mouse induced with the Abelson murine leukemia virus, were selected to investigate the anti-inflammatory effects of AA because they were sensitive to the stimulation of LPS. Additionally, RAW264.7 cells were kindly provided by the Nutritional Immunology Laboratory at PNU (Prof. HM Kim). The cells were cultured in Dulbecco’s Modified Eagle’s medium (DMEM; Thermo Scientific Inc.) containing 10% fetal bovine serum (FBS), 2 mM L-glutamine, 100 U/mL penicillin, and 100 μg/mL streptomycin (Thermo Scientific Inc.). RAW264.7 macrophages were cultured in a humidified incubator at 37 °C under a 5% CO_2_ atmosphere.

To determine the optimal concentration of AA, RAW264.7 macrophages (3 × 10^4^ cells) were seeded into each well of a 96-well plate. When the cells reached 70-80% confluence, they were treated with various concentrations of AA (10, 20, 40, and 80 μM), dissolved in 1× phosphate-buffered saline (PBS) buffer, for 24 h. Supernatants were discarded after incubation for 24 h, followed by the addition of 200 μL of fresh DMEM and 50 μL of 3-(4,5-dimethylthiazol-2-yl)-2,5-diphenyltetrazolium bromide (MTT) solution (20 mg/mL) in 1× PBS to each well, and subsequently incubated at 37 °C for 4 h. Formazan precipitates in the cells were dissolved in DMSO (Duchefa Biochemie), and the absorbance of each well was determined at 570 nm using a Versamax^TM^ microplate reader (Molecular Devices). Based on the above results, the optimal concentrations of AA were determined to be 10, 20, and 40 μM (Supplementary Figure S1).

The optimal concentration of lipopolysaccharide (LPS) was determined using the method used to determine the optimal concentration of AA. After treatment with four concentrations of LPS (0.25, 0.5, 1, and 2 μg/mL) for 24 h, the viability of RAW264.7 macrophages was analyzed with MTT and NO assays. Based on these results, the optimal concentration of LPS was determined to be 1 μg/mL (Supplementary Figures S2 and S3).

To determine the anti-inflammatory effects of AA, RAW264.7 macrophages cultured in the same manner as above were treated with LPS (1 μg/mL) for 1 h and consecutively three concentrations of AA, namely a low (LAA, 10 μM), medium (MAA, 20 μM), and high (HAA, 40 μM) concentration of AA. After further incubation for 24 h, the viability of the cells was measured by a MTT assay, as described above. The Vehicle-treated group received the same volume of DMSO solvent, but the positive (Po)-treated group received only 10 μM of standard AA (BioFront Technologies, Tallahassee, FL, USA). The NO concentration in the supernatant from each cell of the subset group was analyzed. Furthermore, the morphological characteristics of RAW264.7 macrophages for each treatment group were observed at 20× magnification using a light microscope (Leica Microsystems, Wetzlar, Germany).

### 4.7. Measurement of NO Concentration

The level of nitrite, a stable reaction product generated from NO with molecular oxygen, was used as an indicator of NO production. The NO concentration in the culture supernatant of RAW264.7 macrophages was measured using Griess reagent (Invitrogen Co., Carlsbad, CA, USA) as described previously [50]. Briefly, RAW264.7 macrophages were preincubated with LPS (1 μg/mL) for 2 h and then treated with 1× PBS or AA (10, 20, and 40 μM) for 12 h. After collecting the supernatants, 100 μL of each was mixed with 100 μL of modified Griess reagent (Invitrogen Co.) in 96-well plates and incubated for 5 min. The absorbance of each well was measured at 540 nm using a Versamax^TM^ microplate reader (Molecular Devices). A standard curve with increasing concentrations of sodium nitrite was generated in parallel and used for quantification.

### 4.8. Experimental Design of the Animal Study

The PNU Institutional Animal Care and Use Committee (IACUC) reviewed and approved the protocol for the AD animal model study (approval no. PNU-2022-0236). The mice were housed at the PNU-Laboratory Animal Resources (LAR) Center accredited by the Korean Food and Drug Administration (KFDA; unit 000231) and the Association for Assessment and Accreditation of Laboratory Animal Care International (AAALAC International; unit 001525). Male BALB/c mice (8-weeks-old) were purchased from Samtako BioKorea (Osan, Republic of Korea). Drinking water and a standard irradiated chow diet (Samtako BioKorea Co.) were provided ad libitum throughout the experimental period. All mice used in this study were reared under specific pathogen-free conditions (SPF) (50 ± 10% relative humidity and 23 ± 2 °C temperature) in a strict light/dark cycle.

**Figure 14:**
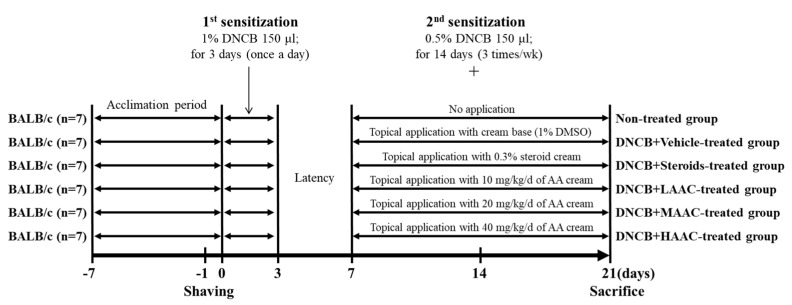
Experimental scheme for AAC spreading onto the skin of DNCB-treated BALB/c mice. Abbreviations: AAC, Abietic acid cream; DNCB, 2,4-Dinitrochlorobenzene.

Briefly, 8-week-old BALB/c mice (n = 42) were assigned to either a non-treated group (n = 7) or a 2,4-dinitrochlorobenzene (DNCB) spread group (n = 35). The second group was further divided into five treatment groups: vehicle cream (DNCB+Vehicle-treated group), steroid cream (LIDOMEX KOWA Ointment 0.3%; Kowa Company Ltd., Nagoya, Japan) (DNCB+Steroids-treated group), low concentration AA cream (DNCB+LAAC-treated group), medium concentration AA cream (DNCB+MAAC-treated group), and high concentration AA cream (DNCB+HAAC-treated group). For the first sensitization, all mice in the DNCB-treated group received a 1% DNCB solution (150 μL) in acetone-olive oil (AOO, 3:1 ratio) for 3 days. After a 4-day stationary phase, 0.5% DNCB solution (150 μL) in AOO was spread onto the dorsal skin three times a week for 14 days to induce a second sensitization. During this period, a cream containing vehicle, steroid or AA was spread on the same DNCB-spread skin region once a day. This cream base was prepared by mixing corn oil (C8267, Sigma-Aldrich Co., St Louis, MO, USA), dH_2_O, and olive wax (Orangeflower, Tokyo, Japan) (20 g: 40 g: 4 g). The DNCB+LAAC-, DNCB+MAAC-, or DNCB+HAAC-treated groups were topically spread with three types of creams containing 10, 20, or 40 mg/kg/d of AA after sufficiently drying the DNCB solution, whereas the DNCB+Steroids-treated group was spread with cream containing 50 μg/kg/day of steroid, which is the reference drugs under identical conditions. The DNCB+Vehicle group received a base cream containing 1% DMSO (Duchefa Biochemie). After the final treatment, mice from all groups were sacrificed with CO_2_ and several tissue samples, including dorsal skin, spleen, and lymph node, were collected for weighting, histopathology, Western blot, and quantitative real-time PCR (RT-qPCR) analyses (Figure 14).

### 4.9. Measurement of Spleen Weight and Lymph Node Size

After collecting spleen and lymph nodes from all mice, the spleen weight was measured using an electronic balance (Mettler Toledo, Greifensee, Switzerland). The size of each lymph node was determined using 64-bit Java 8 (NIH, Madison, WC, USA).

### 4.10. Determination of Dermatitis Score and Skin Thickness

The severity of DNCB-sensitized dorsal skin was evaluated using the SCORing Atopic Dermatitis (SCORAD) index [54]. Dermatitis scores of 0 (no lesion) to 3 (severe) were assigned based on the degree of erythema, edema, papulation, excoriation, and lichenification observed on the dorsal skin. Skin thickness was measured on the last day of the experiment using a thickness gauge (Digimatic Indicator; Matusutoyo Co., Tokyo, Japan).

### 4.11. Histopathological Analysis

After collecting dorsal skin tissue from BALB/c mice, it was fixed using 10% neutral buffered formaldehyde (pH 6.8), dehydrated in an alcohol dilution series, trimmed with a sharp knife, and embedded in paraffin wax. Dorsal skin tissue sections were deparaffinized with xylene (DaeJung Chemicals, Siheung, Republic of Korea) and rehydrated using an alcohol dilution series (100–70%). After washing with distilled water, skin tissues were stained with hematoxylin and eosin (H&E; Sigma-Aldrich Co.) and toluidine blue (TB; Sigma-Aldrich Co.). Histopathological changes were observed at 100× magnification using a Leica application suite (Leica Microsystems). The thickness of the epidermis and dermis as well as the number of mast cells and eosinophils was measured using the Leica Application Suite (Leica Microsystems).

### 4.12. Enzyme-Linked Immunosorbent Assay (ELISA) of IgE

Immunoglobulin E (IgE) concentration in the serum of BALB/c mice was determined using an IgE ELISA kit (Invitrogen Co.), according to the manufacturer’s instructions. Briefly, the same volume (50 μL) of serum samples and standards diluted with a dilution solution was added to antibody-coated wells and subsequently incubated for 2 h at room temperature. The wells were then washed three times with a washing solution (50 mM Tris, 0.14 M NaCl, 0.05% Tween 20, pH 8.0), followed by the addition of 50 μL biotin-conjugated avidin (1000-fold dilution) to each well and incubation for 2 h at room temperature. After washing, horseradish peroxidase-conjugated antibodies (2000-fold dilution) were added to each well and incubated for 1 h at room temperature. The enzyme reaction was initiated by adding tetramethylbenzidine (TMB) substrate solution in the dark for 20 min. Finally, the reaction was terminated by adding an acidic solution (reaction stopper, 2 M H_2_SO_4_), and the absorbance of the yellow product was measured spectrophotometrically at 450 nm using a Versamax^TM^ microplate reader (Molecular Devices).

### 4.13. Western Blot Analysis

Total protein from RAW264.7 macrophages and homogenates of mouse dorsal skin tissues was obtained using Pro-Prep Protein Extraction Solution (iNtRON Biotechnology, Seongnam, Korea) according to the manufacturer’s protocol. After centrifugation at 13,000 rpm for 5 min, the protein concentration of the supernatant was determined using a Pierce^TM^ bicinchoninic acid (BCA) Protein Assay Kit (Thermo Fisher Scientific Inc.). They were separated by 4-20% sodium dodecyl sulfate-polyacrylamide gel electrophoresis (SDS-PAGE) for 2 h, followed by transfer to nitrocellulose membranes (Amersham^TM^ Protran^TM^, GE Healthcare, Chicago, IL, USA) at 40 V for 2 h. Membranes were then incubated overnight at 4 °C with the following primary antibodies: iNOS (Cell Signaling Technology, Danvers, MA, USA), COX-2 (Cell Signaling Technology), SAPK/JNK antibody (Cell Signaling Technology), p-SAPK/JNK (Thr183/Tyr185) antibody (Cell Signaling Technology), p44/42 MAPK (ERK1/2) antibody (Cell Signaling Technology), p-p44/42 MAPK (ERK1/2; Thr202/Tyr204) antibody (Cell Signaling Technology), p38 MAPK antibody (Cell Signaling Technology), p-p38 MAPK (Thr180/Tyr182) antibody (Cell Signaling Technology), NLRP3 (Cell Signaling Technology), ASC/TMS1 (Cell Signaling Technology), Caspase-1 (Cas-1, Cell Signaling Technology), and anti-β-actin antibody (Cell Signaling Technology). Next, the membranes were washed with washing buffer (137 mM NaCl, 2.7 mM KCl, 10 mM Na_2_HPO_4_, and 0.05% Tween 20) and incubated with 1:1000 diluted horseradish peroxidase (HRP)-conjugated goat anti-rabbit IgG (Cell Signaling Technology) at room temperature for 1 h. Finally, membrane blots were developed using the EZ-Western Lumi Femto Kit (Dogen, Seoul, Korea). The chemiluminescence signals originating from specific bands were detected using FluorChemi^®^FC2 (Alpha Innotech, San Leandro, CA, USA).

### 4.14. Quantitative Real-Time-PCR (RT-qPCR) Analysis

RT-qPCR was used to measure iNOS, COX-2, tumor necrosis factor (TNF)-α, IL-1β, IL-4, IL-5, IL-6, and IL-10 mRNA levels as previously described [50]. First, total mRNA was purified from RAW264.7 macrophages and dorsal skin tissues of the subset groups using TRIzol reagent (Favorgen Biotech, Ping-Tung, Taiwan), according to the manufacturer’s protocol. After determining the total RNA concentrations, complementary DNA (cDNA) was synthesized using Superscript II reverse transcriptase (Thermo Fisher Scientific Inc.), and RT-qPCR was performed using the cDNA template (2 μL) and 2× Power SYBR Green (7 μL; Toyobo Life Science, Osaka, Japan) containing specific primers (Supplementary Table S2). RT-qPCR was performed for 40 cycles of denaturation at 95 °C for 15 s, annealing at 70 °C for 60 s, and extension at 70 °C for 60 s. Fluorescence intensities were measured at the end of the extension phase of each cycle. Threshold values for sample fluorescence intensities were set manually and reaction cycles in which the PCR products exceeded these fluorescence intensity thresholds during the exponential phase were considered threshold cycles (Ct). The expression of iNOS, COX-2, TNF-α, IL-1β, IL-4, IL-5, IL-6, and IL-10 was quantified relative to that of the housekeeping gene β-actin, based on a comparison of the Ct values at constant fluorescence intensity [55].

### 4.15. Statistical Analysis

One-way ANOVA was used to determine the statistical significance between the Vehicle+LPS-treated group and the AA+LPS-treated group, as well as between the DNCB+Vehicle-treated group and DNCB+AAC-treated group, and a *p*-value less than 0.05 was reported as statistically significant. All values in the results are presented as mean ± standard deviation (SD).

## 5. Conclusions

**Figure 15:**
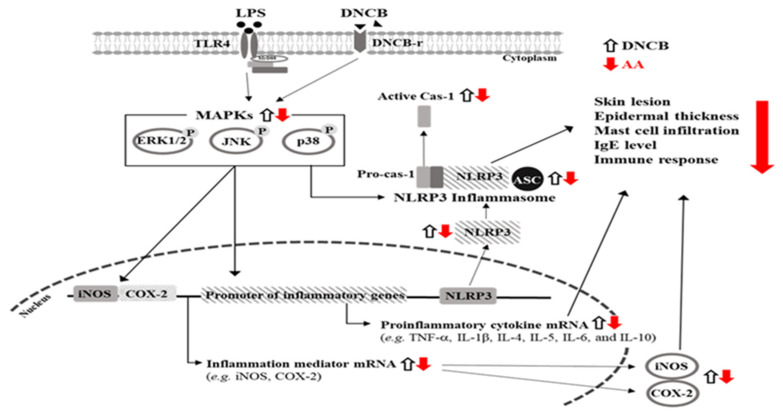
Suggested mechanism for mitigation effects of AA in AD. In this scheme, the treatment of AD is thought to ameliorate the enhancement of skin thickness, dermatitis score, immune organ weight, IgE concentration, and mast cell infiltration through the regulation of iNOS-induced COX-2 mediated pathway and transcription of inflammatory cytokines. Abbreviations: DNCB, 2,4-dinitrochlorobenzene; LPS, lipopolysaccharide; AA, abietic acid; iNOS, inducible nitric oxide synthase; COX-2, cyclooxygenase-2; MAPK, mitogen-activated protein kinase; ERK, extracellular signal-regulated kinase; JNK, c-Jun N-terminal kinase; TNF-α, tumor necrosis factor alpha; IL, interleukin.

In the present study, we attempted to establish the optimal condition for AA isolation as well as to demonstrate the novel therapeutic effects of AA against AD using an animal disease model. To achieve this objective, the conditions for isomerization and crystallization were applied to RSM and CCD analysis, and the anti-inflammatory effects of AA were confirmed in LPS-stimulated RAW264.7 macrophages. The purity and yield of AA from rosin were significantly improved to 99.33% and 58.61% compared to the previous methods under a certain amount of HCl (2.49 mL), reflux extraction time (61.7 min), and ethanolamine (7.35 mL). In addition, anti-AD effects and mechanisms—including the dermatitis score, IgE concentration, skin histopathological structure of the skin, iNOS-induced COX-2 mediated pathway, and the secretion of inflammatory cytokines—were investigated in DNCB-treated BALB/c mice (Figure 15). The results of the present study provide the first scientific evidence that AA has the potential to ameliorate AD symptoms and identify molecular targets related to the condition. Further research involving various disease models is required to understand the mechanisms of action of AA.

## Data Availability

Data is contained within the article and Supplementary Material.

## Supplementary Materials

The following supporting information can be downloaded at https://www.mdpi.com/article/10.3390/ph16030407/s1, Table S1. RSM design to maximize the extraction yield and improve the purity of AA from rosin, Table S2: Primer sequences for RT-qPCR analyses, Figure S1: Cell viability assay to determine the optimal AA concentration, Figure S2: Cell viability assay to determine the optimal LPS concentration, Figure S3: The NO concentration was used to determine the optimal concentration of LPS.
