# Supplementary material for: Anti-Atopic Dermatitis Effects of Abietic Acid Isolated from Rosin under Condition Optimized by Response Surface Methodology in DNCB-Spread BALB/c Mice
Source: Pharmaceuticals (Basel). 2023 Mar 7;16(3):407. doi: 10.3390/ph16030407 (PMC10054120; doi:10.3390/ph16030407)

Supplement Table S1. RSM design to maximize the extraction yield and improve the purity of AA from rosin.

Run no.	The amount of HCl added (mL; X_1)	The reflux extraction time (min; X_2)	The amount of ethanolamine added (mL; X_3)
1	1.01	34.19	4.05
2	2.49	75.81	4.05
3	1.75	55.00	10.00
4	2.49	75.81	15.95
5	3.00	55.00	10.00
6	1.75	55.00	20.00
7	1.75	90.00	10.00
8	0.50	55.00	10.00
9	1.01	75.81	4.05
10	2.49	34.19	15.95
11	1.01	75.81	15.95
12	1.75	20.00	10.00
13	1.75	55.00	0.00
14	1.01	34.19	15.95
15	2.49	34.19	4.05
16	1.75	55.00	10.00
17	1.75	55.00	10.00

Supplement Table S2. Primer sequences for RT-PCR analyses

Primer name	Sequence (from 5' to 3')	Product size (bp)
iNOS		
Forward	CACTT GGAGT TCACC CAGT	169
Reverse	ACCAC TCGTA CTTGG GATGC	
COX-2		
Forward	CAGGT CATTG GTGGA GAGGT GTATC	484
Reverse	CCAGG AGGAT GGAGT TGTTG TAGAG	
TNF-α		
Forward	CCTGT AGCCC ACGTC GTAGC	70
Reverse	TTGAC CTCAG CGCTG ACTTG	
IL-6		
Forward	TTGGG ACTGA TGTTG TTGAC A	200
Reverse	TCATC GCTGT TGATA CAATC AGA	
IL-1β		
Forward	CTACA GGCTC CGAGA TGAAC AAC	79
Reverse	CTACA GGCTC CGAGA TGAAC AAC	
IL-13		
Forward	CCTTA AGGAG CTTAT TGAGG AGCTG AG	280
Reverse	CAGTT GCTTT GTGTA GCTGA GCAG	
IL-4		
Forward	GAATA TACCA GGAGC CATAT C	385
Reverse	CTCAG TACTA CGAGT AATCC A	
IL-10		
Forward	CAGCC GGGAA GACAA TAACT G	67
Reverse	CCGCA GCTCT AGGAG CATGT	
β-actin		
Forward	TGGAA TCCTG TGGCA TCCAT GAAAC	349
Reverse	TAAAA CGCAG CTCAG TAACA GTCCG	

Supplementary Figure S1. Cell viability assay to determine the optimal AA concentration. The MTT assay was performed in RAW264.7, cells after four different doses (10, 20, 40, and 80 μM) of AA. All values in the results are presented as mean ± standard deviation (SD). * indicates statistical significance compared with the non-treated group.

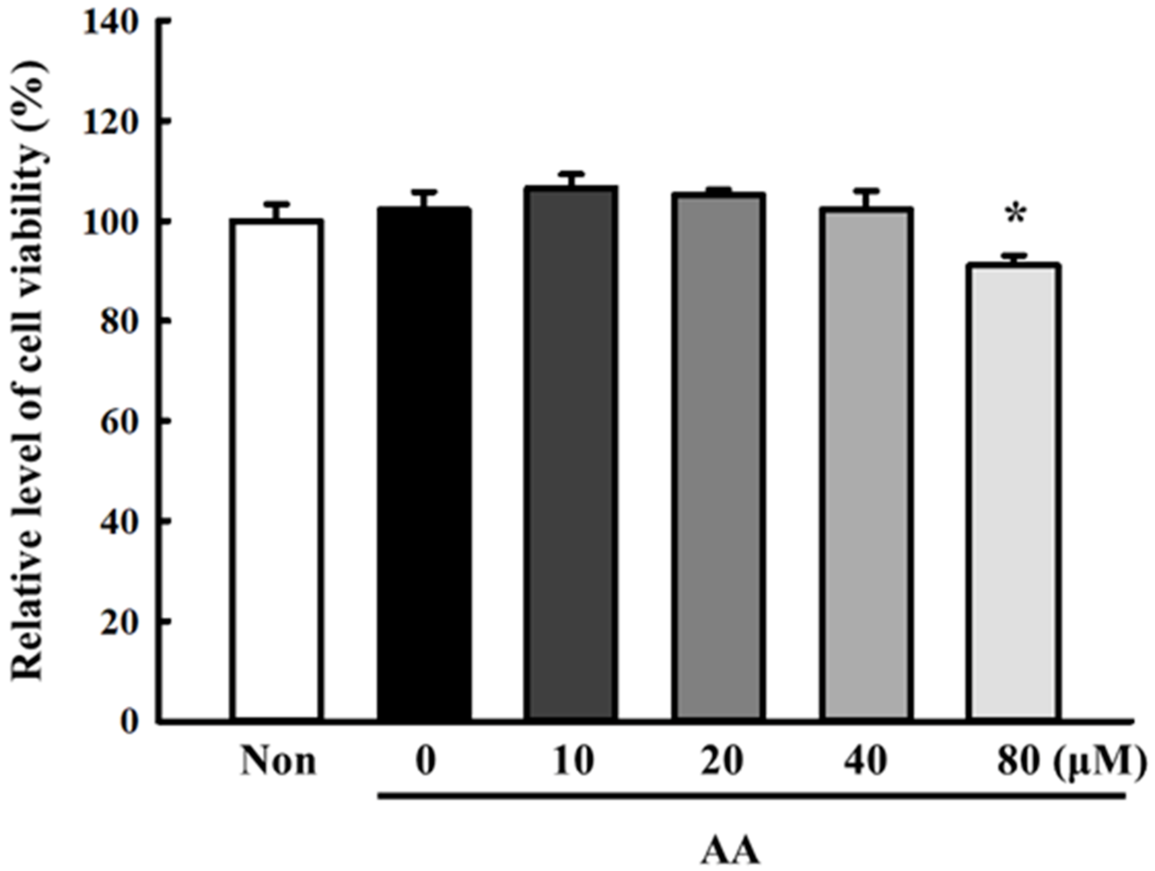

Supplementary Figure S2. Cell viability assay to determine the optimal LPS concentration MTT assay was performed in RAW264.7 cells after four different doses (0.25, 0.5, 1, and 2 μg/mL) of LPS. All values in the results are presented as mean ± standard deviation (SD). * indicates statistical significance compared to the non-treated group.

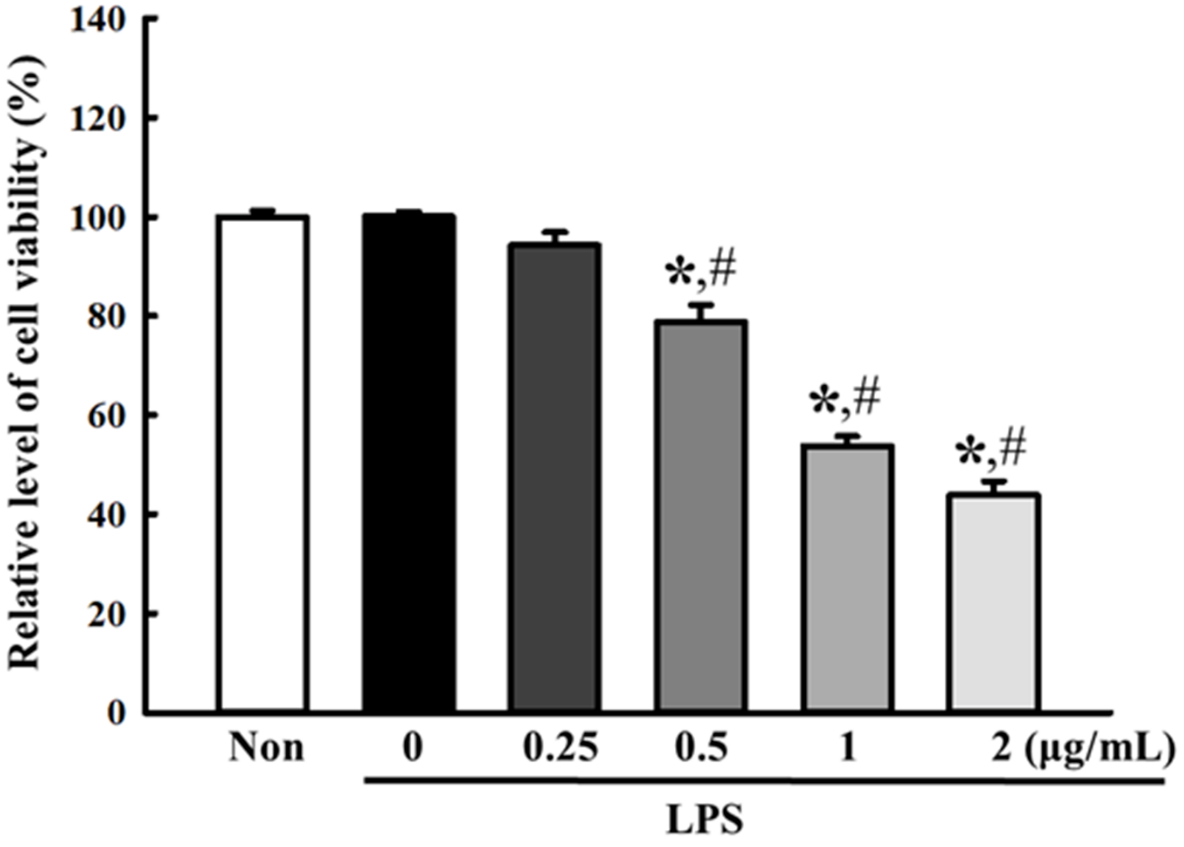

Supplementary Figure S3. The NO concentration was used to determine the optimal concentration of LPS. MTT assay was performed in RAW264.7 cells after four different doses (0.25, 0.5, 1, and 2 μg/mL) of LPS. All values in the results are presented as mean ± standard deviation (SD). * indicates statistical significance compared to the non-treated group.

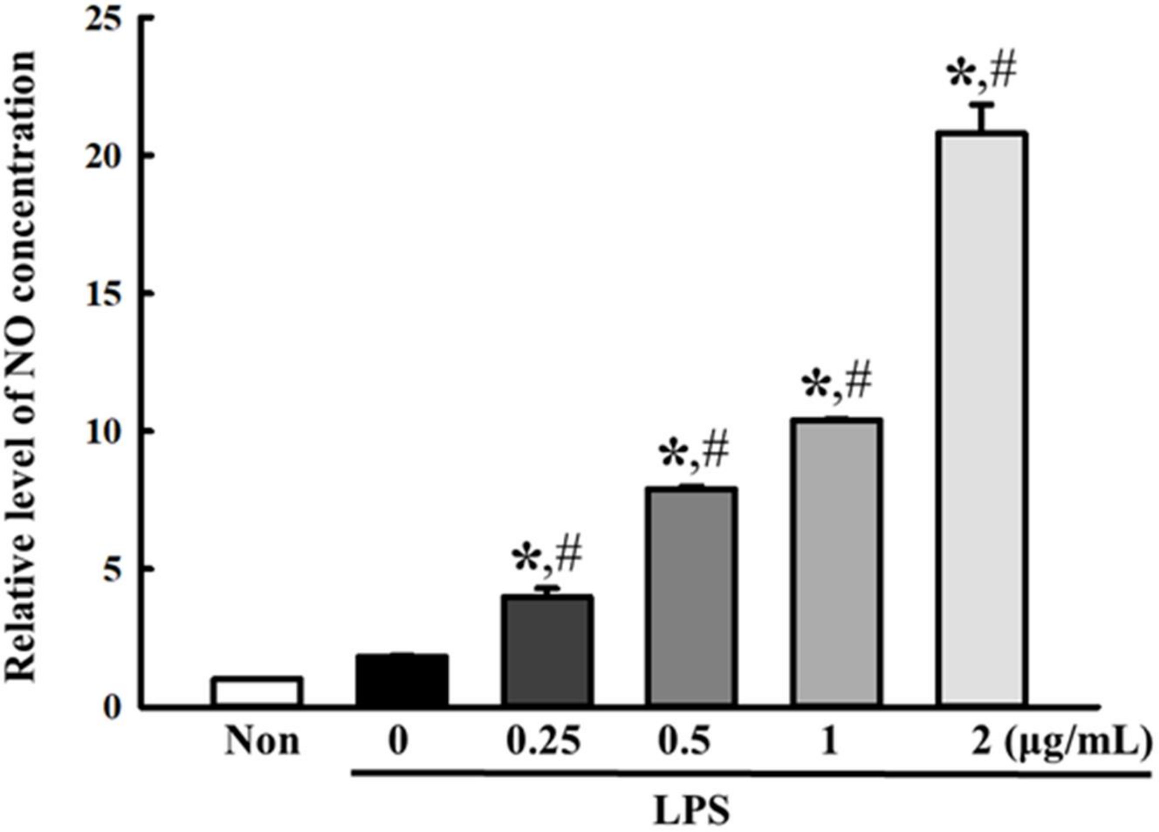

Supplement: Supplementary file 1 [file pharmaceuticals-16-00407-s001.zip › pharmaceuticals-2240666-supplementary.pdf]
